# Effect of Dendrigraft Generation on the Interaction between Anionic Polyelectrolytes and Dendrigraft Poly(l-Lysine)

**DOI:** 10.3390/polym10010045

**Published:** 2018-01-04

**Authors:** Feriel Meriem Lounis, Joseph Chamieh, Laurent Leclercq, Philippe Gonzalez, Jean-Christophe Rossi, Hervé Cottet

**Affiliations:** IBMM, Université de Montpellier, CNRS, ENSCM, 34095 Montpellier, France; lounisferiel@hotmail.fr (F.M.L.); Joseph.chamieh@umontpellier.fr (J.C.); laurent.leclercq@umontpellier.fr (L.L.); Philippe.gonzalez@univ-montp2.fr (P.G.); jean-christophe.rossi@umontpellier.fr (J.-C.R.)

**Keywords:** polyelectrolyte complexes, dendrimers, frontal analysis continuous capillary electrophoresis, counter-ion release, binding constants, ionic strength dependence

## Abstract

In this present work, three generations of dendrigraft poly(l-Lysine) (DGL) were studied regarding their ability to interact with linear poly (acrylamide-*co*-2-acrylamido-2-methyl-1-propanesulfonate) (PAMAMPS) of different chemical charge densities (30% and 100%). Frontal analysis continuous capillary electrophoresis (FACCE) was successfully applied to determine binding constants and binding stoichiometries. The effect of DGL generation on the interaction was evaluated for the first three generations (G2, G3, and G4) at different ionic strengths, and the effect of ligand topology (linear PLL vs. dendrigraft DGL) on binding parameters was evaluated. An increase of the biding site constants accompanied with a decrease of the DGL-PAMAMPS (*n*:1) stoichiometry was observed for increasing DGL generation. The logarithm of the global binding constants decreased linearly with the logarithm of the ionic strength. This double logarithmic representation allowed determining the extent of counter-ions released from the association of DGL molecules onto one PAMAMPS chain that was compared to the total entropic reservoir constituted by the total number of condensed counter-ions before the association.

## 1. Introduction

Dendrimers are nano-sized, radially symmetrical molecules with well-defined and monodisperse structure consisting of tree-like arms or branches [1]. Due to their exceptional architecture, dendrimers have found various applications in supramolecular chemistry, particularly in host–guest reactions and self-assembly processes. They constitute very promising candidates in many biomedical applications because of their possibility to perform controlled and specified drug delivery [2,3,4,5], their use in anticancer therapy [6,7,8] and imaging diagnostic analysis [9,10]. Dendrimers can also be used as solubility enhancers [11,12,13,14], for layer-by-layer deposition [15] and catalysis [16,17,18]. Complexes containing dendrimers and linear polyelectrolytes or (bio)macromolecules have attracted great attention [19,20,21,22,23]. Some experimental and theoretical investigations were interested in determining size and structural properties of linear-dendritic polyelectrolyte complexes [22,24,25,26,27,28,29,30]. However, little is known about the influence of ramification on the thermodynamic binding parameters (stoichiometry, binding constant, enthalpy, entropy) of such polyelectrolyte complexes (PEC). 

Giri et al. [19] studied the binding of human serum albumin (HSA) and poly (amidoamine) (PAMAM) dendrimers. Binding constants gradually increased with dendrimer generation (from 1.67 × 10^5^ M^−1^ for G0 to 5.42 × 10^6^ M^−1^ for G6) followed by a slight decrease for G8 dendrimer (3.3 × 10^6^ M^−1^). This study showed that binding constants depended on the chemical structure of the core and the terminal group of dendrimers. Furthermore, DNA-PAMAM dendrimer complex stability and binding constant were found to increase with dendrimer generation [31]. Kabanov et al. [28] showed that the complexes of poly(propylene imine) dendrimers with DNA or synthetic linear polyanions containing equal amounts of cationic and anionic groups were stoichiometric and insoluble in water. Water-soluble non-stoichiometric complexes were obtained when dendrimers were introduced in default in the complex. 

Dendrigraft poly-l-lysine (DGL) are dendritic synthetic cationic polypeptides synthesized by successive polycondensation of *N*-trifluoroacetyl-l-lysine-*N*-carboxyanhydride in water. Compared to dendrimers, DGL have a linear core (and not point core) and more flexible structures [32]. In previous investigations, frontal analysis continuous capillary electrophoresis (FACCE) was found to be a straightforward method to study interactions between dendrigraft poly(l-Lysine) (DGL) (G3) and oppositely charged biomolecules such as adenosine monophosphate (AMP), adenosine triphosphate (ATP) ligands [21], and human serum albumin (HSA) [33,34]. HSA-G3 interactions studies under physiological conditions, demonstrated that HSA had two cooperative binding sites with G3 with the following successive constants *K*_1_ = 31.2 × 10^3^ M^−1^ and *K*_2_ = 30.6 × 10^3^ M^−1^. Increasing DGL generation (G1 to G5) led to an increase of the binding constant accompanied with a decrease of the HSA:DGL (1:*n*) stoichiometry and a decrease of the cooperativity with dendrimer generation [33]. 

In the present work, we propose to study the effect of DGL generation and the influence of the polylysine topology by comparison with a linear poly-l-Lysine (PLL) on polyelectrolyte complexes (PEC) formation. The interaction between DGL (G2–G4) (or linear PLL) and statistical copolymers of acrylamide and 2-acrylamido-2-methyl-1-propanesulfonate (PAMAMPS) with chemical charge densities of 30% and 100%, were investigated by FACCE. The DGL-PAMAMPS interactions and PLL-PAMAMPS interactions are discussed in term of stoichiometry, binding constants, and amount of released counter-ions during the complex formation. This study represents a quantitative investigation of how the ionic strength, the chemical charge density and the topology (linear vs. dendritic) of polyelectrolytes influence the thermodynamic binding parameters when PEC are formed. This works also brings new experimental data about oppositely charged macromolecules, including the experimental estimation of condensed and released counter-ions, which constitutes a topic of interest from a theoretical point of view [35,36].

## 2. Materials and Methods

### 2.1. Chemicals

Random copolymers of acrylamide and 2-acrylamido-2-methyl-1-propanesulfonate (PAMAMPS) with chemical charge densities *f* of 30% and 100% were synthesised by free radical copolymerization as described by McCormick et al. [37]. The details of the synthesis are reported elsewhere [38] and briefly described in the Supplementary. *DP*_w_ of PAMAMPS 30% (respectively 100%) were 3689 (respectively 4166) as obtained by Size-Exclusion Chromatography Coupled with Multi-Angle Laser Light Scattering (SEC-MALLS) and published elsewhere [38]. Molar mass distributions obtained by SEC-MALLS and charge density (or chemical composition) distributions obtained by capillary electrophoresis are available in supporting information of a previously published study [38]. Average chemical composition was also confirmed by ^1^H NMR [38]. Poly-l-Lysine (PLL) (with a degree of polymerization *DP*_n_ = 50, corresponding to a molar masse of 8200 g/mol and a polydispersity index 1.04) was supplied by Alamanda Polymers (Huntsville, AL, USA). DGL (G2, G3, and G4) (batch numbers; DC 120902, DC 120103, DC 130604, respectively) were supplied by Colcom (Montpellier, France). Polydiallyldimethylammonium chloride (PDADMAC), *M*_w_ = 400–500 kDa, ammonium bicarbonate and sodium azide were purchased from Sigma Aldrich (St Quentin Fallavier, France). Tris(hydroxymethyl)aminomethane (Tris, (CH_2_OH)_3_CNH_2_, 99.9%) was purchased from Merck (Darmstadt, Germany). Hydrochloric acid 37%, sodium hydroxide and sodium chloride were purchased from VWR (Leuven, Belgium). 2-2Bis(hydroxymethyl)-2,2′,2′′-nitrilotriethanol (Bis Tris, 99%) was purchased from Acros Organics (Geel, Belgium). Cellulose ester dialysis membrane of 100 Da (reference number: 131 018) was purchased from Spectrum Labs (Rancho Dominguez, CA, USA). Durapore^©^ membrane filters were purchased from Merck Millipore (Darmstadt, Germany). Deionised water was further purified using a Milli-Q system (Millipore, Molsheim, France). All chemical (except DGL) were used without any further purification.

### 2.2. Intergeneration Purification of DGL by Semi-Preparative Size-Exclusion Chromatography

Intergeneration purification of DGL was realised by semi-preparative size exclusion chromatography (SEC) in order to remove any residual DGL molecules of the previous generations or any residual salts. SEC purifications were carried out on an Äkta purifier 100 GE healthcare system (Vélizy-Villacoublay, France) with a UV detector set at 210 nm. A Superdex 200 column (30 cm × 1 cm) with an exclusion domain between 1000 and 100,000 Da was used for DGL intergeneration separation. The particles of this column had a diameter between 22 and 44 µm. The mobile phase was ammonium bicarbonate 0.1 M at pH 11 at a flow rate of 1 mL/min. DGL samples were prepared at 30 g/L in mobile phase, and 500 µL were manually injected. The purified fractions were evaporated to eliminate the excess of the eluent and then freeze-dried. For DGL-PAMAMPS interactions, purified DGL were dissolved in a 10^−4^ M HCl solution, dialysed against the same HCl solution and then freeze-dried.

### 2.3. Measurement of DGL Refractive Index Increments

Before carrying out the refractive index measurements, a 2 g/L mother solution of each purified DGL generation dissolved in the eluent was dialysed against the eluent using a cellulose ester dialysis membrane of 100 Da. The eluent was the same for dialysis, refractive index increment measurements and molar mass distribution experiments. It was composed of 50 mM Bis Tris, 1 M sodium chloride, 0.3 g/L sodium azide. pH was adjusted to 6 using 1 M hydrochloric acid solution. The eluent was finally filtrated using Durapore membrane filters (0.1 µm cutoff, Millipore, Molsheim, France). The refractive index increments (d*n*/d*C*) of different DGL generations were determined at 35 °C using a Shimadzu RID-6A (Tokyo, Japan) refractive detector set at 690 nm. The instrument was calibrated using sodium chloride solutions of various known concentrations (2.0, 1.5, 1.0, 0.75, 0.5 and 0.25 g/L) to get a d*n*/d*C* value of 0.187. DGL solutions were prepared at 2.0, 1.5, 1.0, 0.75, 0.5 and 0.25 g/L by diluting the dialysed mother solution with the eluent. A volume of 2 mL of each DGL solution was injected into the refractometer. The refractive index increments (d*n*/d*C*) were calculated using the Astra software (v6.1.1.17, Wyatt Technology Corp., Santa Barbara, CA, USA).

### 2.4. Determination of the Molar Mass Distribution of DGL by Size-Exclusion Chromatography Coupled with Multi-Angle Laser Light Scattering (SEC–MALLS)

The weight-average molar masses (*M*_w_) and polydispersity indexes (*PDI*) of purified DGL were determined using size-exclusion chromatography coupled with multi-angle laser light scattering (SEC–MALLS). Dialyzed samples at 2 g/L of DGL in the eluent (see previous section) were eluted using a Thermo Scientific Ultimate module 3000 separations at a flow rate of 0.8 mL/min equipped with column guard SHODEX OHpak SBG (50 × 6 mm) and two columns SHODEX SB-806M-HQ (300 × 8 mm) (Munich, Germany) connected in series. The eluent used for SEC-MALLS analyses was the same as described in the previous section. The eluted samples were detected using a mini DAWN-TREOS three-angles (45°, 90°, 135°) laser light scattering detector with a laser at 690 nm (Wyatt Technology Corp., Santa Barbara, CA, USA) and a RID-6A refractive index monitor Shimadzu (Tokyo, Japan) at a thermostated temperature of 35 °C. The data for molar mass determination were analysed using ASTRA software (v6.1.1.17, Wyatt Technology Corp., Santa Barbara, CA, USA).

### 2.5. Preparation of Polyelectrolytes Mixtures

PAMAMPS, PLL and DGL stock solutions were prepared in 12 mM Tris, 10 mM HCl and NaCl buffer (pH 7.4) at room temperature. The ionic strength of the buffer was adjusted by adding adequate amounts of NaCl. The concentrations of PAMAMPS stock solutions were 2 and 1.14 g/L for PAMAMPS 30% and 100%, respectively. The concentration of PLL and DGL stock solutions was 5 g/L. Diluted PLL and DGL solutions with concentrations from 0.1 to 4 g/L for polyelectrolytes mixtures and from 0.1 to 2 g/L for calibration curves, were prepared by dilution in the same Tris-HCl-NaCl buffer. PLL-PAMAMPS and DGL-PAMAMPS mixtures were prepared by adding 100 µL of the polyanionic stock solutions to 100 µL of the polycationic solutions (see Tables S1 and S2 for the concentrations of PAMAMPS, PLL, and DGL in the mixtures). The final mixtures with a volume of 200 µL were equilibrated by homogenizing with a vortex stirrer during 1 min. DGL-PAMAMPS mixtures were incubated for 12 h and then analysed by FACCE.

### 2.6. FACCE Procedure

Capillary electrophoresis experiments were carried out using an Agilent 3D system (Agilent, Waldbronn, Germany). Separations were realized using bare fused silica capillaries from Polymicro Technologies (Photonlines, Saint-Germain-en-Laye, France). Capillary dimensions were 50 µm internal diameter (i.d.) × 33.5 cm total length (8.5 cm to the detector). New capillaries were flushed for 30 min with a 1.0 M NaOH solution and with water for 20 min. The capillary inner surfaces were then coated with polycationic polymer by flushing the capillary for 20 min with a 0.2% *w*/*w* poly diallyldimethylammonium chloride (PDADMAC) solution prepared in a 2 M NaCl solution. Before each run the capillary was flushed with water for 2 min, PDADMAC 0.2% *w*/*w* in water for 3 min and finally Tris-HCl-NaCl buffer for 3 min. To reduce the migration times, samples were placed at the capillary end, which is the closest to the detection point (8.5 cm). The temperature of the capillary was kept at 25 °C and the detection wavelength was set at 200 nm. FACCE experiments were achieved by applying a continuous positive polarity voltage of +1 kV (from the injection end) and a *co*-pressure of 5 mbar (from the injection end) for PLL molecules and 4 mbar for DGL molecules, in order to allow the continuous electrokinetic entry of the free ligand molecules (free DGL or free PLL) contained in the equilibrated mixtures. These voltage and pressure conditions allowed the selective entry and the quantification of the free ligand preventing the entry of free PAMAMPS and PEC molecules. 

## 3. Results and Discussions

### 3.1. Characterization of Purified DGL

Intergeneration purity of DGL turned out to be important in order to make accurate measurements of binding parameters between the DGL and the linear polyanions. For that reason, each DGL generation was purified by semi-preparative SEC as described in Section 2.2 and further analysed by SEC-MALLS analysis for the determination of molar mass distribution. In SEC analysis, the separation of DGL was exclusively governed by size exclusion. The refractive index increments were measured for each DGL-Cl generation after dialysis against the eluent to ensure that the chemical potential is constant in the eluent and in the injected DGL solution (see Section 2.3). Relatively narrow molar mass distributions were obtained (see Figure S1 in the Supplementary Materials for the molar mass distributions of the purified DGL). Table 1 reports the values of the refractive index increment (d*n*/d*C*), the weight-average molar mass (*M*_w_) and the polydispersity index (*PDI*) for each DGL-Cl generation. It is known that, for any dendritic structure including DGL, the molar mass increases exponentially with increasing dendrimer generation number [33], which explains the high increase of the molar mass with the increase in generation number. The weight-average degree of polymerization *DP*_w_ was obtained by dividing the weight-average molar masses (*M*_w_) by the average molar mass of the monomer (*M*_0_) taking into account the fraction of condensed counter-ions [39] according to:

(1) $$M_{0}=\left(M_{\mathrm{lys}}\times \left(1-\theta^{+}\right)\right)+\left(M_{{\mathrm{lys},\mathrm{Cl}}^{-}}\times \theta^{+}\right)$$

where *M*_lys_ is the molar mass of a lysine residue (*M*_lys_ = 129 g/mol), *M*_lys,Cl_^−^ is the molar mass of a lysine residue + Cl^−^ counter-ion (*M*_lys,Cl_^−^ = 164.5 g/mol), θ^+^ is the fraction of condensed counter-ions.

### 3.2. Determination of DGL-PAMAMPS Binding Parameters by FACCE

**Figure 1:**
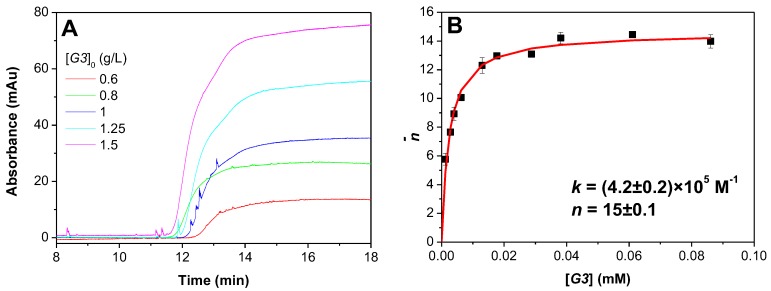
Example of determination of binding parameters (*n* and *k*) by FACCE for the interaction G3-PAMAMPS 30% at 552 mM ionic strength. Electropherograms obtained for different G3-PAMAMPS 30% equilibrated mixtures (**A**); and the corresponding isotherm of adsorption (**B**) representing the number of bound G3 entities per PAMAMPS 30% chain according to the free G3 concentration. Experimental conditions: PDADMAC coated capillary 33.5 cm (8.5 cm to the detector) × 50 µm i.d. Background electrolyte: 12 mM Tris, 10 mM HCl, 542 mM NaCl, pH 7.4. Applied voltage + 1 kV with a *co*-hydrodynamic pressure of +4 mbar. Detection at 200 nm. Samples were prepared in the background electrolyte by 50/50 *v*/*v* dilution of the following solutions: PAMAMPS 30% at 2 g/L with G3 at 5, 4, 3, 2.5, 2, 1.6, 1.2, 1, 0.8, 0.6 g/L.

It is obvious that the interactions between DGL and PAMAMPS are expected to be highly dependent on the DGL generation and very different compared to poly(l-lysine)/PAMAMPS interactions, due to important changes in polycation topology. To examine the effect of dendrimer generation on the thermodynamic binding parameters, isotherms of adsorption were plotted for three successive generations of DGL (G2, G3, and G4) in interaction with linear polyanions of different charge densities (PAMAMPS 30% and PAMAMPS 100%). In this work, we have used a PAMAMPS 30% (instead of 15% in our former publication about PLL/PAMAMPS interactions [41]) because DGL/PAMAMPS 15% mixtures required very long equilibrium times (more than 24 h), while in the case of the PDGL/PAMAMPS 30% the equilibrium was reached in less than 12 h. For that purpose, FACCE methodology developed by Sisavath et al. [34] was used. In this method, a continuous voltage and a *co*-pressure were simultaneously applied in order to introduce selectively the free ligand (free DGL) in the capillary, avoiding the dynamic dissociation of the complex during electrophoretic migration. As a result, the free ligand continuously entering in the capillary is detected as a plateau, the height of which is proportional to the free ligand concentration at equilibrium in the mixture. The free ligand concentrations were determined using a calibration curve performed for each DGL generation in the same electrophoretic conditions. Examples of electropherograms obtained by FACCE for DGL G3 in the presence of PAMAMPS 30%, at 552 mM ionic strength are given in Figure 1A. Isotherms of adsorption were plotted by representing the average number of bound ligands (DGL) per substrate molecule (PAMAMPS) $\bar{n}$ (calculated according to Equation (2)) vs. the free ligand concentration [*DGL*] for different initial molar ratio [*DGL*]_0_/[*PAMAMPS*]_0_. The stoichiometry of interaction *n* expressed in term of bound DGL entities per PAMAMPS chain and the binding site constant *k* were determined by non-linear least square routine on Microsoft excel using the model of identical interacting sites [42] expressed by Equation (3).

(2) $$\bar{n}=\frac{{\left[DGL\right]}_{0}-[DGL]}{{\left[PAMAMPS\right]}_{0}}$$

(3) $$\bar{n}=\frac{nk[DGL]}{1+k[DGL]}$$

where [*DGL*]_0_ and [*DGL*] are the initial and free ligand concentrations, respectively. [*PAMAMPS*]_0_ is the concentration of PAMAMPS initially introduced in the mixture. It is worth noting that the intrinsic binding constant *k* refers to the association of one ligand (one DGL molecule) onto a binding site –*s* corresponding to a small portion of PAMAMPS:

(4) $$-s+DGL\overset{k}{⇄}DGL-s$$

(5) $$k=\frac{\left[DGL-s\right]}{\left[DGL][-s\right]}$$

where [*DGL*] and [*DGL*− *s*] are the free and complexed DGL concentrations at equilibrium respectively, [− *s*] is the concentration of free sites. 

An example of isotherm of adsorption for the interaction G3-PAMAMPS 30% at 552 mM ionic strength is given in Figure 1B (see Figures S2−S7 in the Supplementary Materials for the display of all the isotherms of adsorption for the other DGL generations and ionic strengths).

**Figure 2:**
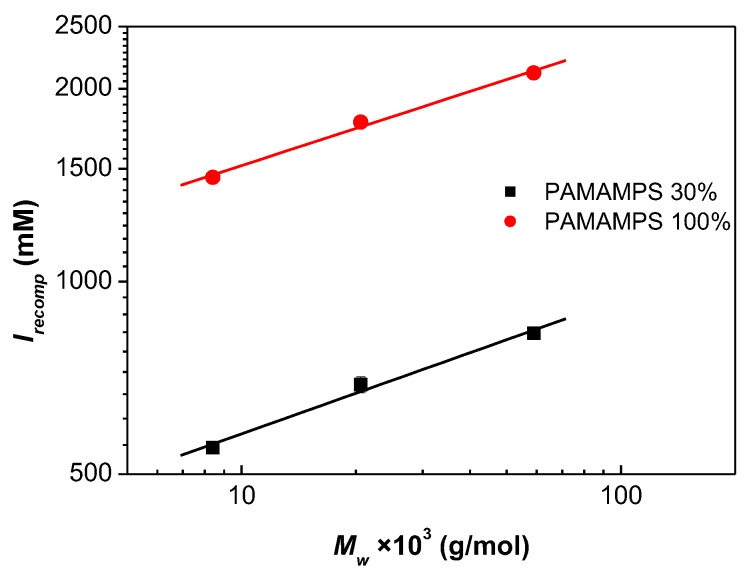
Variation of the ionic strength of recomplexation *I*_recomp_ as a function of the molar mass of the purified DGL.

In order to get the ionic strength dependence of the binding constants and stoichiometries, isotherms of adsorption were determined at different ionic strengths *I* for each DGL generation and for two PAMAMPS chemical charge density (30% and 100%). However, accurate measurements of the binding parameters by FACCE required the adjustment of the ionic strengths to avoid unmeasurable high binding constants at low ionic strength, or low affinities due to the dissociation of PEC at high ionic strength. For that, the ionic strength of recomplexation *I*_recomp_ (defined as the salt concentration at which a solid DGL-PAMAMPS complex previously destabilized at high ionic strength re-formed when water was added) was first determined by turbidimetry, as previously described [38]. Figure 2 shows the variation of *I*_recomp_ according to the molar masses of the purified DGL in double logarithmic scale. As can be seen in Figure 2, the logarithm of the salt concentration required to dissociate a DGL-PAMAMPS complex varied linearly with the logarithm of the DGL molar masses. The binding parameters *n* and *k* were measured in a range of ionic strength from 40% to 90% of *I*_recomp_ (i.e., between 220–747 mM for the interactions DGL-PAMAMPS 30%; and between 870–2011 mM for the interactions DGL-PAMAMPS 100%) yielding measurable binding site constants *k* between 1.6 × 10^4^ M^−1^ and 7.7 × 10^6^ M^−1^.

### 3.3. Influence of the Ionic Strength and DGL Generation on the Stoichiometry of Interaction

**Figure 3:**
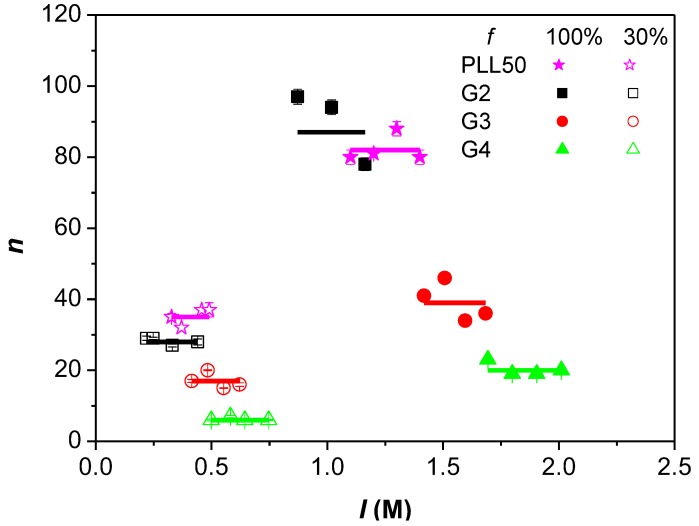
Variation of the interaction stoichiometry *n_(_*_Lys/AMPS)_, expressed as the number of DGL molecules per PAMAMPS chain, as a function of the ionic strength for the interactions between DGL (or PLL50) with PAMAMPS 30% and PAMAMPS 100%.

The stoichiometry of interactions *n*_(*DGL/PAMAMPS*)_ expressed in term of bound DGL molecules per PAMAMPS chain at saturation of the isotherms (i.e., in the presence of an excess of DGL) is presented in Figure 3. The ionic strength did not significantly influence the binding stoichiometry whatever the PAMAMPS chemical charge density and the DGL generation number. This result is similar to what was observed in a previous work for linear PLL [41,42,43]. The fluctuations in stoichiometry were attributed to experimental errors. It was found that the stoichiometry *n*_(*DGL/PAMAMPS*)_ decreased when the generation number of DGL increased, in good agreement with the increase in molar mass, as observed for linear poly(l-lysine) [41]. 

The charge stoichiometry expressed in terms of lysine residues per AMPS monomers (*n*_(Lys/AMPS)_) is given in Table 2 and Table 3 for PAMAMPS 30% and 100%, respectively. As for PAMAMPS 30%, *n*_(Lys/AMPS)_ is higher than one whatever the DGL generation number, and also for PLL 50. This is in good agreement with the general rule recently enounced [38] stating that, when the highest charged polyelectrolyte partner (PLL or DGL in that case) are introduced in excess, then the formed PEC has a stoichiometry in favour of the highest charged polyelectrolyte (here the polycation, i.e., *n*_(Lys/AMPS)_ > 1). DGL G2 and the linear PLL50 (*DP* 50) have almost the same molar masses (8400 and 8200 g/mol, respectively). It is thus interesting to consider the effect of the ramification on the PEC stoichiometry at comparable molar masses. Interestingly, the charge stoichiometry was, in average, slightly lower for DGL G2 (1.31) than for PLL50 (1.56), as if the charge parameter of DGL G2 was lower than for PLL50. On the other hand, for DGL G3 and G4, the *n*_(Lys/AMPS)_ stoichiometry was higher than for PLL50, as if the DGL G3 and G4 were more charged compared to PLL50. Even if the charge parameter is hardly accessible for the DGL, or even not well-defined, one can compare the counter-ion condensation rates θ^+^ that were previously determined by isotachophoresis [40], as an indication of the polyelectrolyte charge density (the higher the condensation rate, the higher the chemical charge density or charge parameter). Finally, it was observed that the charge stoichiometry correlated well with the counter-ion condensation rate, as shown in Table 2.

As for the PLL50/PAMAMPS 100% interaction, the polyanion is the highest charged polyelectrolyte and it is introduced in default at saturation of the isotherm. In that case, the rule states that the charge stoichiometry tends to 1 [38], as observed experimentally for that system (*n*_(Lys/AMPS)_ = 0.99). In the case of the DGL/PAMAMPS 100%, the *n*_(Lys/AMPS)_ stoichiometry seems to be higher than one, and seems to increase with the increase in generation number, as if the charge density (or charge parameter) of the DGL increased with the DGL generation and becomes higher than the PAMAMPS 100% linear charge density. Again, these results correlate well with the increase of the counter-ion condensation with the DGL generation, i.e., an increase of the charge parameter with the DGL generation.

### 3.4. Influence of the Ionic Strength and DGL Generation on the Binding Constants

**Figure 4:**
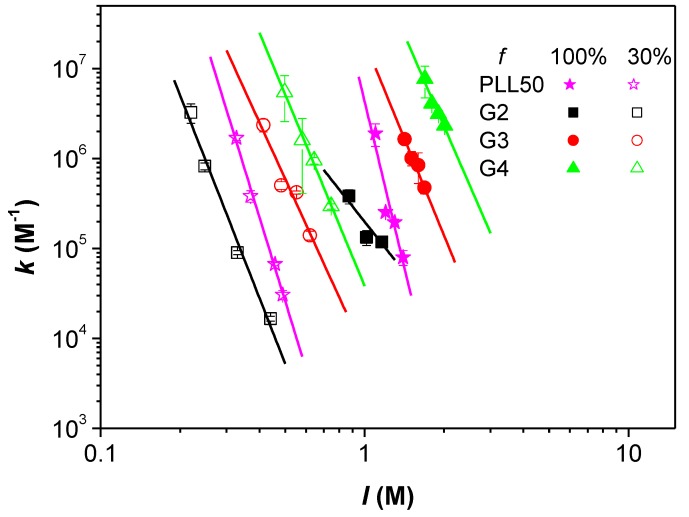
Variation of the binding site constant *k* as a function of the ionic strength *I* for the interactions between DGL (or PLL50) with PAMAMPS 30% and PAMAMPS 100%.

According to the model of identical interacting sites previously described for the study of PLL/PAMAMPS interactions [41,42], the binding site constant *k* represents the equilibrium constant relative to the interaction between one DGL molecule (one ligand) with one interacting site –*s* of a PAMAMPS chain (substrate). This interacting site –*s* is defined as a fragment of PAMAMPS chain carrying electrostatic anchoring AMPS monomers. The variation of binding site constants *k* with the ionic strength is represented in Figure 4, where log *k* decreased linearly with log *I*. Moreover, for a given ionic strength, the higher the generation is, the higher the binding constant is, for both DGL-PAMAMPS 30% and DGL-PAMAMPS 100% systems. This increase of binding site constant can be qualitatively explained by the increase of the number of possible electrostatic anchoring points on each ligand, which grows exponentially with increasing DGL generation number. 

The global binding constant *β_n_*, representing the equilibrium associated to the full binding of the *n* sites of the substrate [42] is related to the binding site constant by Equation (6)

(6) $$\beta_{n}=k^{n}$$

**Figure 5:**
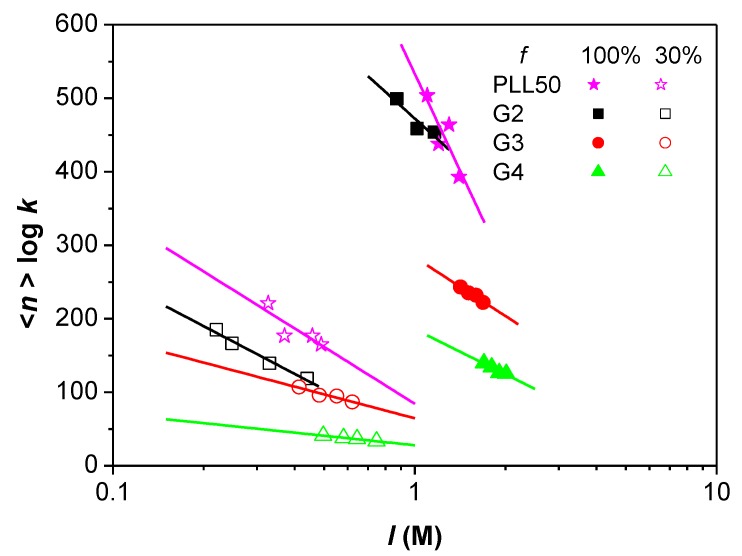
Variation of log *β_n_* as a function of log *I* for the interactions between DGL (or PLL50) with PAMAMPS 30% and PAMAMPS 100%.

As observed for PLL/PAMAMPS systems [41,42,43] and for other systems in the literature [45,46,47,48,49,50,51,52,53], the logarithm of *β_n_* was found to decrease linearly with the ionic strength of the medium (see Figure 5) with an experimental slope representing the number of the counter-ions effectively released during the formation of one PEC composed of one PAMAMPS chain and *n* ligands. This double logarithmic dependence was attributed to the entropically dominant character of oppositely charged polyelectrolytes interactions. In Figure 5, log *β_n_* was calculated using log *β_n_* = <*n*> *×* log *k*, where <*n*> is the average value of stoichiometry on the four ionic strengths investigated for each DGL (or PLL50). It can be seen from Figure 5, that the interaction of PAMAMPS with higher dendrimer generations is less sensitive to the ionic strength than lower generations and linear PLL, this observation is in perfect agreement with the computer simulations in the literature [54].

**Figure 6:**
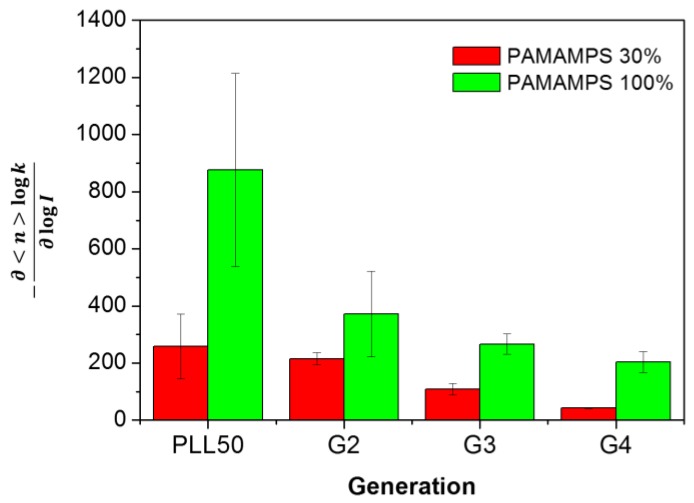
Variation of the number of counter-ions effectively released ($-\frac{\partial <n>\log k}{\partial \log I}$) for the linear PLL50 and for DGL G2, G3 and G4 in interaction with PAMAMPS 30% or PAMAMPS 100%.

**Figure 7:**
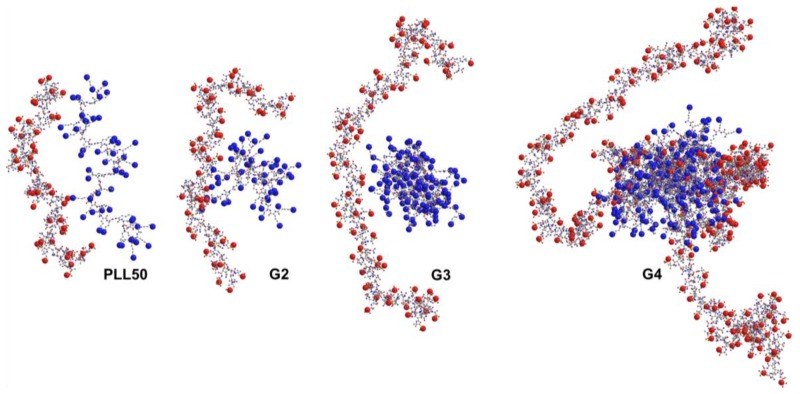
Schematic representation of the interaction between one ligand (PLL or DGL molecule) and one binding site –*s* of a PAMAMPS 30% chain. The *DP* of one binding site on the PAMAMPS substrate is: 128, 160, 265 and 750 for PLL50, G2, G3 and G4, respectively, as calculated by dividing the *DP* of PAMAMPS 30% by the stoichiometry *n*_(PLL or_ *_DGL/PAMAMP_*_)_ of the interaction. Red dots represent the negative charge of the sulfonate groups and blue dots represent the positive charge of the ammonium groups.

The number of counter-ions that are effectively released from the association of *n* ligands onto one PAMAMPS chain can be determined by calculating $-\frac{\partial <n>\log k}{\partial \log I}$ [41,42,43]. These numerical values are reported in Table 2 and Table 3 for PAMAMPS 30% and 100% respectively, and are compared to the one obtained for linear PLL50-PAMAMPS interactions (see Figure 6). It can be observed that the higher the DGL generation number is, the lower the number of counter-ions effectively released is. For all DGL generations, the number of released counter-ions is lower than that observed for PLL50. It was previously observed for linear PLL of different molar masses that the number of released counter-ions decreases with increasing PLL molar mass, due to the formation of longer PAMAMPS loops in the PEC structure. In the case of DGL, the decrease of the number of released counter-ions may be due to the non-accessibility of the PAMAMPS chain into the DGL structure which may become even more dramatic for higher DGL generation. Figure 7 displays a schematic image of the interaction on one binding site in the case of PAMAMPS 30%, using the charge stoichiometry determined in this work. The *DP* of one binding site on the PAMAMPS 30% chain increased from 128 to 160 when going from linear PLL (*DP* = 50) to DGL 2nd generation, G2 (*DP*_n_ = 48), and increased with increasing DGL generation, 265 for G3 (*DP*_n_ = 123) and 750 for G4 (*DP*_n_ = 365). It clearly illustrates the steric constraints for the PAMAMPS chain to access the core of the DGL, especially for higher generations. Thus, despite the increase of the total number of condensed counter-ions before the association (*N*_counter-ions_) given in Table 2 and Table 3, (from ~4800 for PLL50 up to ~8500 for DGL G4 with PAMAMPS 100%; and from ~900 for PLL50 up to ~1700 for DGL G4 with PAMAMPS 30%), this huge entropic reservoir is not effectively released after the association. To better illustrate this effect, the percentage of released counter-ions compared to the total number of counter-ions condensed on both polyelectrolyte partners, decreases when the DGL generation number increases (from 38% to 3% for DGL-PAMAMPS 30% interactions, and from 8% to 2% for DGL-PAMAMPS 100% interactions). It is worth noting that, in this work, and as a first approximation, *N*_counter-ions_ was estimated via the Manning condensation theory. Therefore, we did not consider here the possible variation of the number of condensed counter-ions according to the ionic strength. This topic is still discussed as some authors reported a decrease of the fraction of condensed counter-ions with an increase of the ionic strength using numerical simulations [55], while others predicted an increase of the condensed counter-ion fraction with increasing ionic strength [56].

## 4. Conclusions

FACCE method was applied to determine the binding parameters between linear and dendrigraft poly(l-Lysine) interacting with linear PAMAMPS. The influence of the ligand topology and the ionic strength on the interactions was studied. At a given ionic strength, it was found that higher DGL generations led to stronger binding site constants. DGL-PAMAMPS (*n*:1) stoichiometries decreased with the increase in DGL generation number and were almost independent of the ionic strength. Furthermore, a double logarithmic linear dependence between the global biding constants and the ionic strength was observed. The slope of each line is a direct experimental estimation of the number of released counter-ions from the association of *n* DGL molecules onto PAMAMPS chains. Surprisingly, the number of released counter-ions decreased with increasing generation number. This result was far from being intuitive since the entropic reservoir, corresponding to the number of initially condensed counter-ions, increases with increasing DGL generation. The fact that only a small proportion of the initially condensed counter-ions are released can be explained by a low accessibility of the PAMAMPS chain to the core of the DGL.

## Figures and Tables

**Table 1:** Refractive index increment (d*n*/d*C*), weight-average molar masses (*M*_w_) and polydispersity index (*PDI*) determined by SEC-MALLS for each purified DGL generation. *M*_0_ is the average molar mass of a lysine monomer taking into account thefractionof cendensed counter-ions (see Equation (1)) and θ^+^ is the fraction of condensed counter-ions [40]. Complete molar mass distributions are given in Figure S1.

Generation	θ^+^	*M*_0_ (g/mol)	*DP*_n_	*DP*_w_	Counter-Ion	d*n*/d*C*	*M*_w_ (g/mol)	*PDI*
G2	0.35	141	57	60	Cl^−^	0.1756	8400	1.05
G3	0.65	152	136	137		0.1989	20723	1.01
G4	0.76	156	367	378		0.1749	58916	1.03

**Table 2:** Physico-chemical properties of oppositely charged polyelectrolytes (DGL G2 G3 or G4, PLL50 and PAMAMPS 30%) and the corresponding parameters of interaction obtained by FACCE. All these parameters were obtained by curve fitting of the isotherms of adsorption (see Figure S2–S4 in the Supplementary Materials for the isotherms of adsorption).

*f* (%) ^a^	θ^− b^	*N_Na_*^+ c^	Polycation	*DP_n_* ^d^	θ^+ e^	*I* (M)	*n*_(*DGL/PAMAMPS*)_	<*n*> ^f^	*n*_(Lys/AMPS)_	<*n*> ^g^	*N_counter-ions_* ^h^	$-\frac{\partial \langle n\rangle log\;k}{\partial log\;I}$	*Average% of Released Counter-Ions*
30	0	0	PLL50	50	0.5	0.327	35	35	1.60	1.56	887	258 ± 113	29 ± 3
						0.37	32		1.42		792		
						0.458	37		1.56		916		
						0.49	37		1.66		919		
			G2	57	0.35	0.22	29	28	1.35	1.31	584	215 ± 21	38 ± 4
						0.25	29		1.36		587		
						0.33	27		1.24		537		
						0.44	28		1.3		560		
			G3	136	0.65	0.41	17	17	1.93	1.96	102	108 ± 20	7 ± 2
						0.48	20		2.45		1735		
						0.55	15		1.64		1290		
						0.62	16		1.82		1420		
			G4	367	0.76	0.5	6	6	1.92	1.97	1653	43 ± 2	3 ± 0.2
						0.58	7		2.12		1827		
						0.64	6		1.94		1673		
						0.75	6		1.89		1632		

^a^ The *DP_w_* of PAMAMPS 30% is 3689; ^b^ The fraction of condensed charged monomers on PAMAMPS 30% chain; see reference [44]; ^c^ The number of Na^+^ counter-ions condensed onto a PAMAMPS 30% chain calculated as in reference [42]; ^d^ The degree of polymerisation of polycations; ^e^ The fraction of condensed charged monomers on PLL or DGL molecules; see reference [40]; ^f^ The average interactions stoichiometry expressed in term of PLL or DGL molecules bound per PAMAMPS chain; ^g^ The average interactions stoichiometries expressed in term of lysine residues bound per AMPS monomers; ^h^ The total entropic reserve of initially condensed counter-ions calculated as in reference [42].

**Table 3:** Physico-chemical properties of oppositely charged polyelectrolytes (DGL G2 G3 or G4, PLL50 and PAMAMPS 100%) and the corresponding parameters of the interactions obtained by FACCE. All these parameters were obtained by curve fitting of the isotherms of adsorption (See Figure S5 to S7 in the Supplementary Materials for the isotherms of adsorption).

*f* (%) ^a^	θ^− b^	*N_Na_*^+ c^	Polycation	*DP_n_* ^d^	θ^+ e^	*I* (M)	*n*_(*DGL/PAMAMPS*)_	<*n*> ^f^	*n*_(Lys/AMPS)_	<*n*> ^g^	*N _counter-ions_* ^h^	$-\frac{\partial \langle n\rangle log\;k}{\partial log\;I}$	*Average% of Released Counter-Ions*
100	0.67	2794	PLL50	50	0.5	1.1	80	82	1.01	0.99	4801	876 ± 338	18 ± 2
						1.2	81		0.97		4820		
						1.3	88		1.05		4988		
						1.4	80		0.96		4799		
			G2	57	0.35	0.87	97	89	1.29	1.11	4722	372 ± 150	8 ± 3
						1.02	94		1.12		4674		
						1.16	78		0.94		4342		
			G3	136	0.65	1.42	41	39	1.23	1.18	6403	226 ± 36	4 ± 1
						1.51	46		1.37		6827		
						1.6	34		1.07		5842		
						1.68	36		1.06		5940		
			G4	367	0.76	1.69	23	20	1.96	1.75	9153	204 ± 37	2 ± 0.5
						1.80	19		1.63		8071		
						1.91	19		1.65		8132		
						2.01	20		1.74		8440		

^a^ The *DP*_w_ of PAMAMPS 100% is 4166; ^b^ The fraction of condensed charged monomers on PAMAMPS 100% chain; see reference [44]; ^c^ The number of Na^+^ counter-ions condensed onto a PAMAMPS 100% chain calculated as in reference [42]; ^d^ The degree of polymerisation of polycations; ^e^ The fraction of condensed charged monomers on PLL or DGL molecules; see reference [40]; ^f^ The average interactions stoichiometry expressed in term of PLL or DGL molecules bound per PAMAMPS chain; ^g^ The average interactions stoichiometries expressed in term of lysine residues bound per AMPS monomers; ^h^ The total entropic reserve of initially condensed counter-ions calculated as in reference [42].

## Supplementary Materials

The following are available online at www.mdpi.com/2073-4360/10/1/45/s1.
