# Supplementary material for: Effect of Dendrigraft Generation on the Interaction between Anionic Polyelectrolytes and Dendrigraft Poly(l-Lysine)
Source: Polymers (Basel). 2018 Jan 4;10(1):45. doi: 10.3390/polym10010045 (PMC6415173; doi:10.3390/polym10010045)
Supplement: Supplementary file 1 [file polymers-10-00045-s001.pdf]

Supporting Information to

Effect of dendrigraft generation on the interaction between anionic polyelectrolytes and dendrigraft poly(L-lysine)

Feriel Meriem Lounis[1], Joseph Chamieh[1], Laurent Leclercq[1], Philippe Gonzalez[1], Jean-Christophe Rossi[1], Hervé Cottet[1*]

[1] IBMM, Université de Montpellier, CNRS, ENSCM, Montpellier 34095, France
[*] Correspondence: herve.cottet@umontpellier.fr; Tel.: (+33) 4 67 14 34 27

1. Intergeneration purification of DGL

Figure S1 represents the molar mass distributions of the purified DGL obtained by SEC-MALLS analysis. Experimental conditions are described in sections 2.3 and 2.4 of the main article.

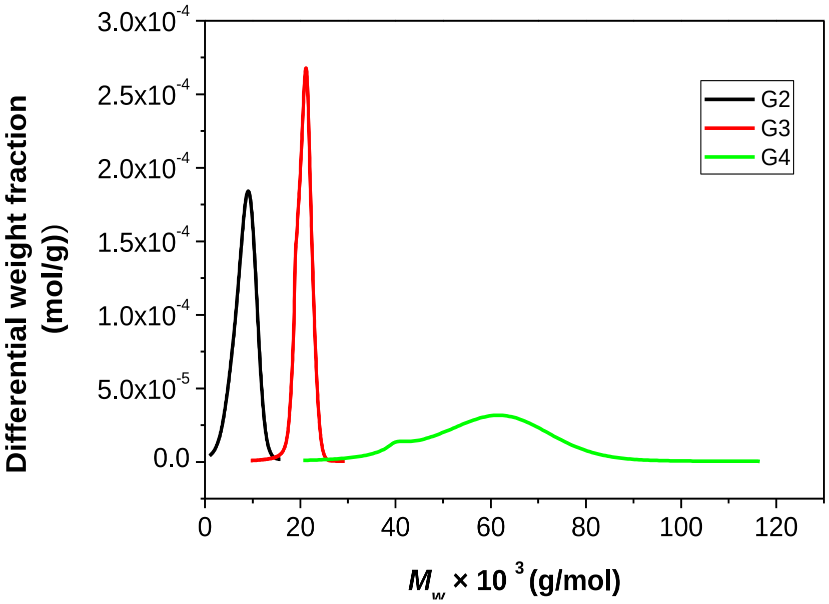

Figure S1. Molar mass distributions of purified DGL (G2, G3 and G4) obtained by SEC-MALLS analysis.

2. Preparation of DGL-PAMAMPS mixtures

The concentrations of the oppositely charged polyelectrolytes for the preparation of DGL-PAMAMPS mixtures as well as the investigated ionic strengths are summarised in Table S1 and Table S2.

Table S1. Concentrations of oppositely charged polyelectrolyte solutions for the preparation of DGL-PAMAMPS 30% mixtures.

DGL	*I* (M)	[*PAMAMPS*] stock solution (g/L)[*]	[*DGL*] stock solution (g/L)[*]	[*PLL*] diluted solutions (g/L)[*]	$V_{PAMAMPS}$ (µL) [(a)]	V_{DGL} (µL) [(b)]	V_f (µL) [(c)]
G2	0.22	2	5	1.25, 1, 0.8, 0.6, 0.5, 0.4, 0.3	100	100	200
	0.25			2; 1.5; 1.25; 1, 0.8, 0.6, 0.5, 0.4, 0.3			
	0.33			2; 1.5; 1.25; 1, 0.8, 0.6, 0.5, 0.4, 0.3; 0.2			
	0.44			2; 1.5; 1.25; 1, 0.8, 0.6, 0.5, 0.4, 0.3; 0.2; 0.1			
G3	0.41			2; 1.5; 1.25; 1, 0.8, 0.6, 0.5, 0.4			
	0.48			2; 1.5; 1.25; 1, 0.8, 0.6, 0.5, 0.4, 0.3			
	0.55			2; 1.5; 1.25; 1, 0.8, 0.6, 0.5, 0.4, 0.3			
	0.62			2; 1.5; 1.25; 1, 0.8, 0.6, 0.5, 0.4, 0.3; 0.2			
G4	0.5			2; 1.5; 1.25; 1, 0.8, 0.6, 0.5			
	0.58			2; 1.5; 1.25; 1, 0.8, 0.6, 0.5			
	0.64			2; 1.5; 1.25; 1, 0.8, 0.6, 0.5, 0.4, 0.3			
	0.75			2; 1.5; 1.25; 1, 0.8, 0.6, 0.5, 0.4, 0.3; 0.2			

(*) For each FACCE experiment, PAMAMPS and DGL stock solutions as well as DGL diluted solutions were prepared in the same Tris-HCl-NaCl buffer pH 7.4 (12mM Tris, 10mM HCl and appropriate amount of NaCl to adjust the ionic strength of the medium). [(a)] Volume of PAMAMPS stock solution in the mixtures [(b)] Volume of DGL diluted solutions in the mixtures [(c)] final volume of the mixtures.

Table S2. Concentrations of oppositely charged polyelectrolyte solutions for the preparation of DGL-PAMAMPS 100% mixtures.

DGL	*I* (M)	[*PAMAMPS*] stock solution (g/L)[*]	[*DGL*] stock solution (g/L)[*]	[*PLL*] diluted solutions (g/L)[*]	$V_{PAMAMPS}$ (µL) [(a)]	V_{DGL} (µL) [(b)]	V_f (µL) [(c)]
G2	0.87	2	5	2; 1.5; 1.25; 1, 0.8, 0.6, 0.5, 0.4, 0.3	100	100	200
	1.02			2; 1.5; 1.25; 1, 0.8, 0.6, 0.5, 0.4, 0.3			
	1.16			2; 1.5; 1.25; 1, 0.8, 0.6, 0.5, 0.4, 0.3			
G3	1.42			1.5; 1.25; 1, 0.8, 0.6, 0.5, 0.4, 0.3			
	1.51			1.5; 1.25; 1, 0.8, 0.6, 0.5, 0.4			
	1.60			1.5; 1, 0.8, 0.6, 0.5, 0.4, 0.3			
	1.68			1.5; 1.25; 1, 0.6, 0.5, 0.4; 0.3; 0.2			
G4	1.69			2; 1.5; 1.25; 1, 0.8, 0.6			
	1.80			2; 1.5; 1.25; 1, 0.8, 0.6; 0.5			
	1.91			2; 1.5; 1.25; 1, 0.8, 0.6; 0.5; 0.4; 0.3; 0.2			
	2.01			1.25; 1, 0.8, 0.6; 0.5; 0.4; 0.3; 0.2			

(*) For each FACCE experiment, PAMAMPS and DGL stock solutions as well as DGL diluted solutions were prepared in the same Tris-HCl-NaCl buffer pH 7.4 (12mM Tris, 10mM HCl and appropriate amount of NaCl to adjust the ionic strength of the medium). [(a)] Volume of PAMAMPS stock solution in the mixtures [(b)] Volume of DGL diluted solutions in the mixtures [(c)] final volume of the mixtures.

3. Isotherms of adsorption

The influence of the ionic strength, the chemical charge density and DGL generation number on the thermodynamic parameters of the interactions between DGL and PAMAMPS was studied by plotting 23 isotherms of adsorption. These isotherms are displayed in Figure S1 to Figure S6 with the non-linear curve fitting allowing the determination of the binding site constants and the stoichiometries expressed in term of DGL molecules bound per PAMMAPS chain.

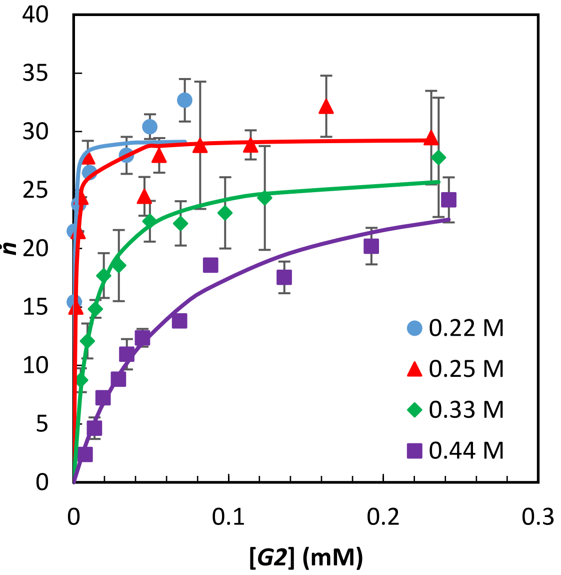

Figure S2. Isotherms of adsorption and non-linear curve fitting obtained for the interactions between G2 and PAMAMPS 30% at different ionic strengths as noticed on the graph. Experimental conditions: PDADMAC coated capillary 33.5cm (8.5 cm to the detector) × 50 µm i.d. Background electrolyte: 12 mM Tris, 10 mM HCl and appropriate amount of NaCl, pH 7.4. Applied voltage: +1 kV with a co-hydrodynamic pressure of +4 mbar. Detection wavelength 200 nm. Samples were prepared in the background electrolyte by 50/50 v/v dilution of PAMAMPS 30% and DGL solutions according to Table S1.

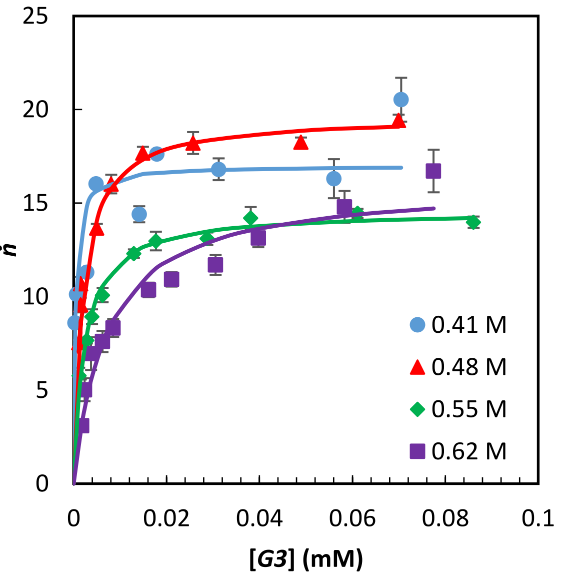

Figure S3. Isotherms of adsorption and non-linear curve fitting obtained for the interactions between G3 and PAMAMPS 30% at different ionic strengths as noticed on the graph. Experimental conditions were the same as in Figure S2.

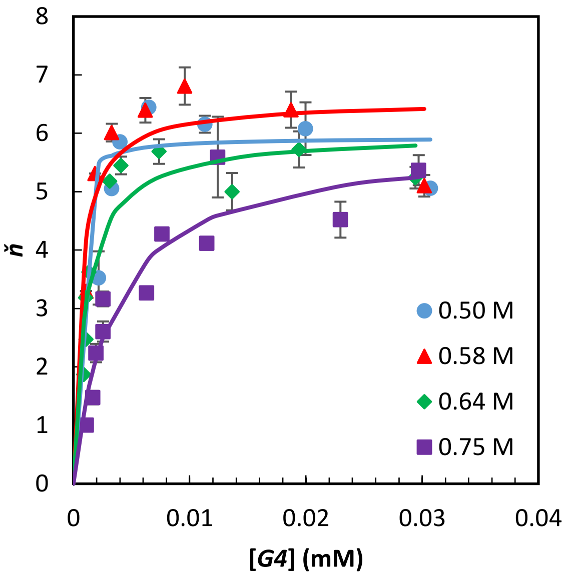

Figure S4. Isotherms of adsorption and non-linear curve fitting obtained for the interactions between G4 and PAMAMPS 30% at different ionic strengths as noticed on the graph. Experimental conditions were the same as in Figure S2.

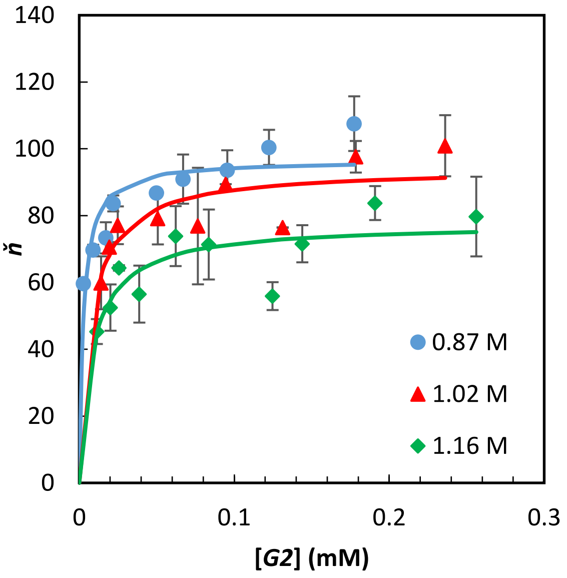

Figure S5. Isotherms of adsorption and non-linear curve fitting obtained for the interactions between G2 and PAMAMPS 100% at different ionic strengths as noticed on the graph. Samples were prepared in the background electrolyte by 50/50 v/v dilution of PAMAMPS 100% and DGL solutions according to Table S2. Other conditions as in Figure S2.

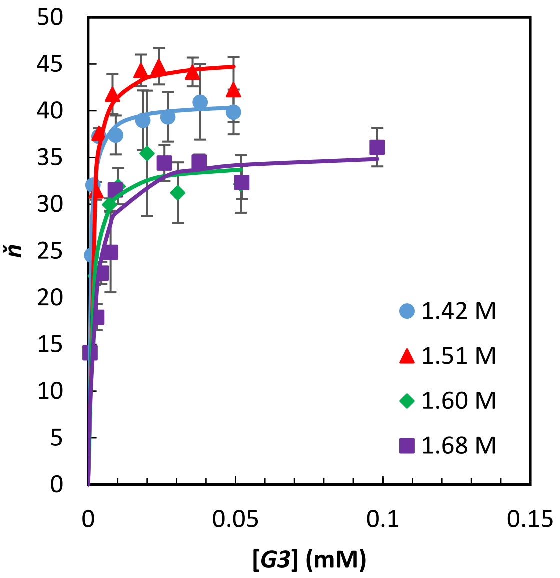

Figure S6. Isotherms of adsorption and non-linear curve fitting obtained for the interactions between G3 and PAMAMPS 100% at different ionic strengths as noticed on the graph. Experimental conditions were the same as in Figure S5.

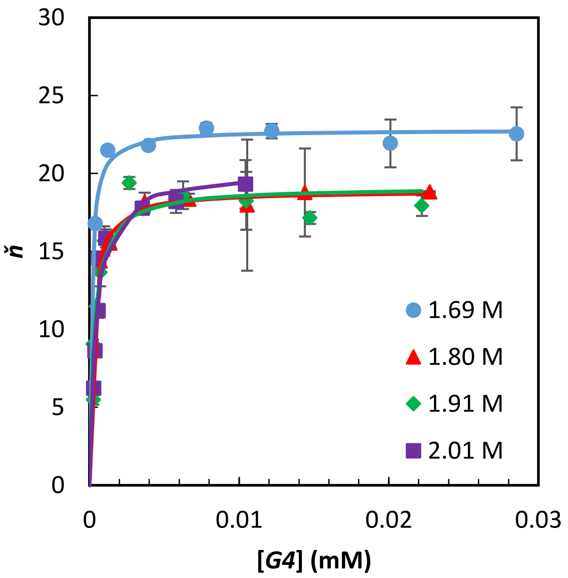

Figure S7. Isotherms of adsorption and non-linear curve fitting obtained for the interactions between G4 and PAMAMPS 100% at different ionic strengths as noticed on the graph. Experimental conditions were the same as in Figure S5.
